# Supplementary material for: Discovery of ZrCoBi based half Heuslers with high thermoelectric conversion efficiency
Source: Nat Commun. 2018 Jun 27;9:2497. doi: 10.1038/s41467-018-04958-3 (PMC6021448; doi:10.1038/s41467-018-04958-3)
Supplement: Supplementary file 1 — Supplementary Information [file 41467_2018_4958_MOESM1_ESM.pdf]

Supporting information

Discovery of ZrCoBi based half Heuslers with high thermoelectric conversion efficiency

Hangtian Zhu[§1], Ran He[§1,2], Jun Mao[§1,3], Qing Zhu[1], Chunhua Li[4], Jifeng Sun[5], Wuyang Ren[1,6], Yumei Wang[7], Zihang Liu[1], Zhongjia Tang[8], Andrei Sotnikov[2], Zhiming Wang[6], David Broido[4], David J. Singh[5], Gang Chen[9], Kornelius Nielsch[2], and Zhifeng Ren[1]*

[1] *Department of Physics and Texas Center for Superconductivity, University of Houston, Houston, TX 77204, U.S.A.*

[2] *Institute for Metallic Materials, IFW-Dresden, Dresden 01069, Germany*

[3] *Department of Mechanical Engineering, University of Houston, Houston, TX 77204, U.S.A.*

[4] *Department of Physics, Boston College, Chestnut Hill, MA 02467, U.S.A.*

[5] *Department of Physics and Astronomy, University of Missouri, Columbia, MO 65211, U.S.A.*

[6] *Institute of Fundamental and Frontier Sciences, University of Electronic Science and Technology of China, Chengdu 610054, China*

[7] *Beijing National Laboratory for Condensed Matter Physics, Institute of Physics, Chinese Academy of Sciences, P.O. Box 603, Beijing 100190, China*

[8] *Department of Chemistry, University of Houston, Houston, TX 77204, U.S.A.*

[9] *Department of Mechanical Engineering, Massachusetts Institute of Technology, Cambridge, MA 02139, U.S.A.*

[§]Equal contributor

[*]Author to whom correspondence should be addressed: zren@uh.edu.

Supplementary Figures

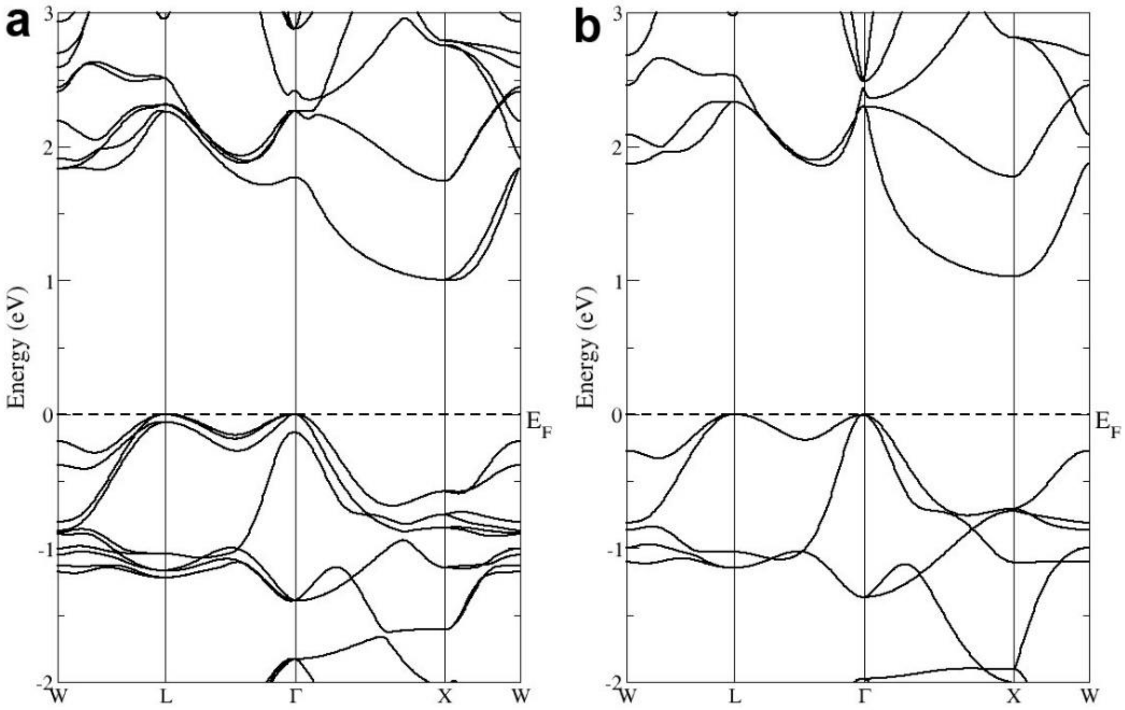

Supplementary Figure 1. Band structure of ZrCoBi. Calculated band structure of ZrCoBi with spin-orbit coupling effect (a) and without spin-orbit coupling effect (b).

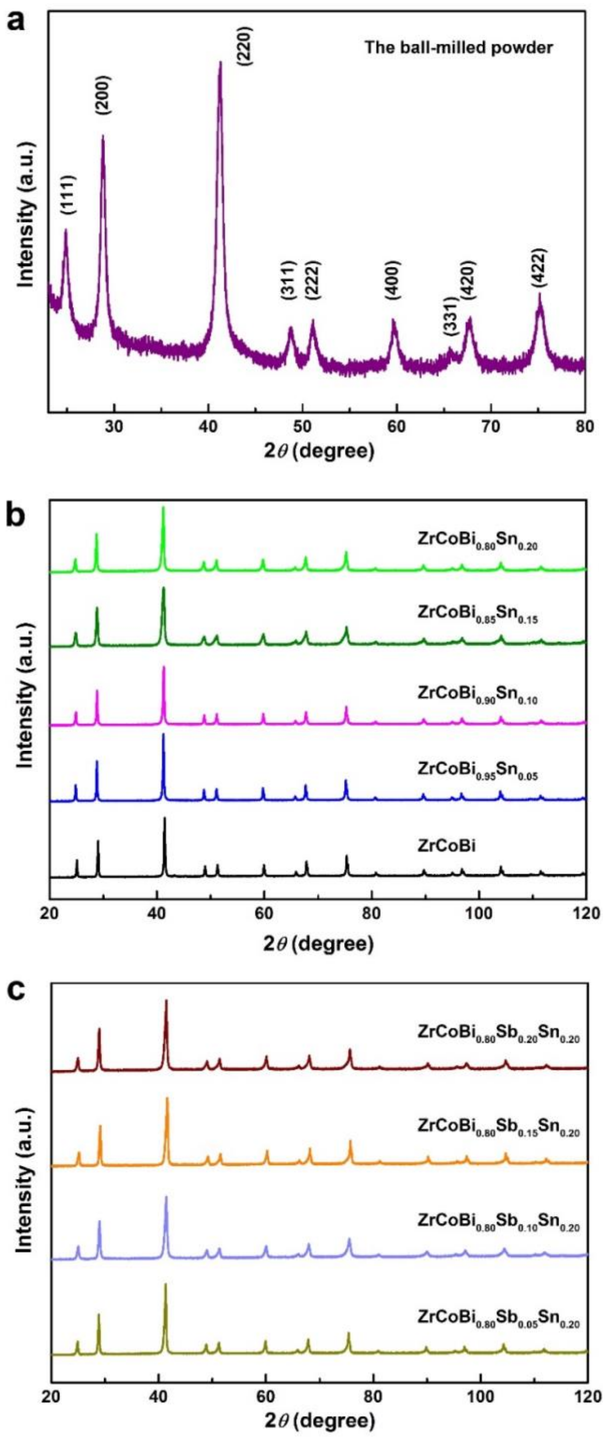

Supplementary Figure 2. Phase characterization. X-ray diffraction patterns of ball-milled ZrCoBi powders (a) and hot-pressed $ZrCoBi_{1-x-y}Sb_ySn_x$ pellets (b-c).

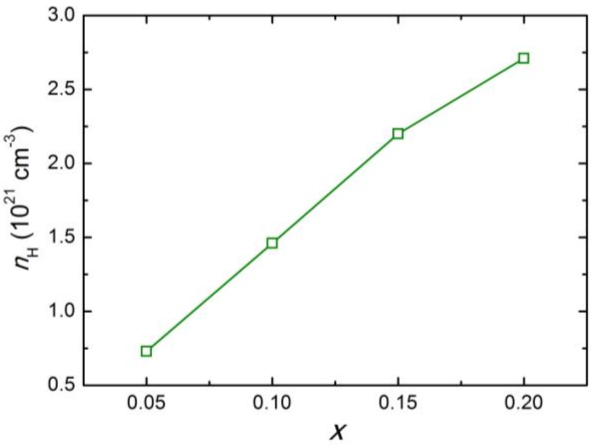

Supplementary Figure 3. Hall carrier concentration. Composition-dependent Hall carrier concentration of $ZrCoBi_{1-x}Sn_x$.

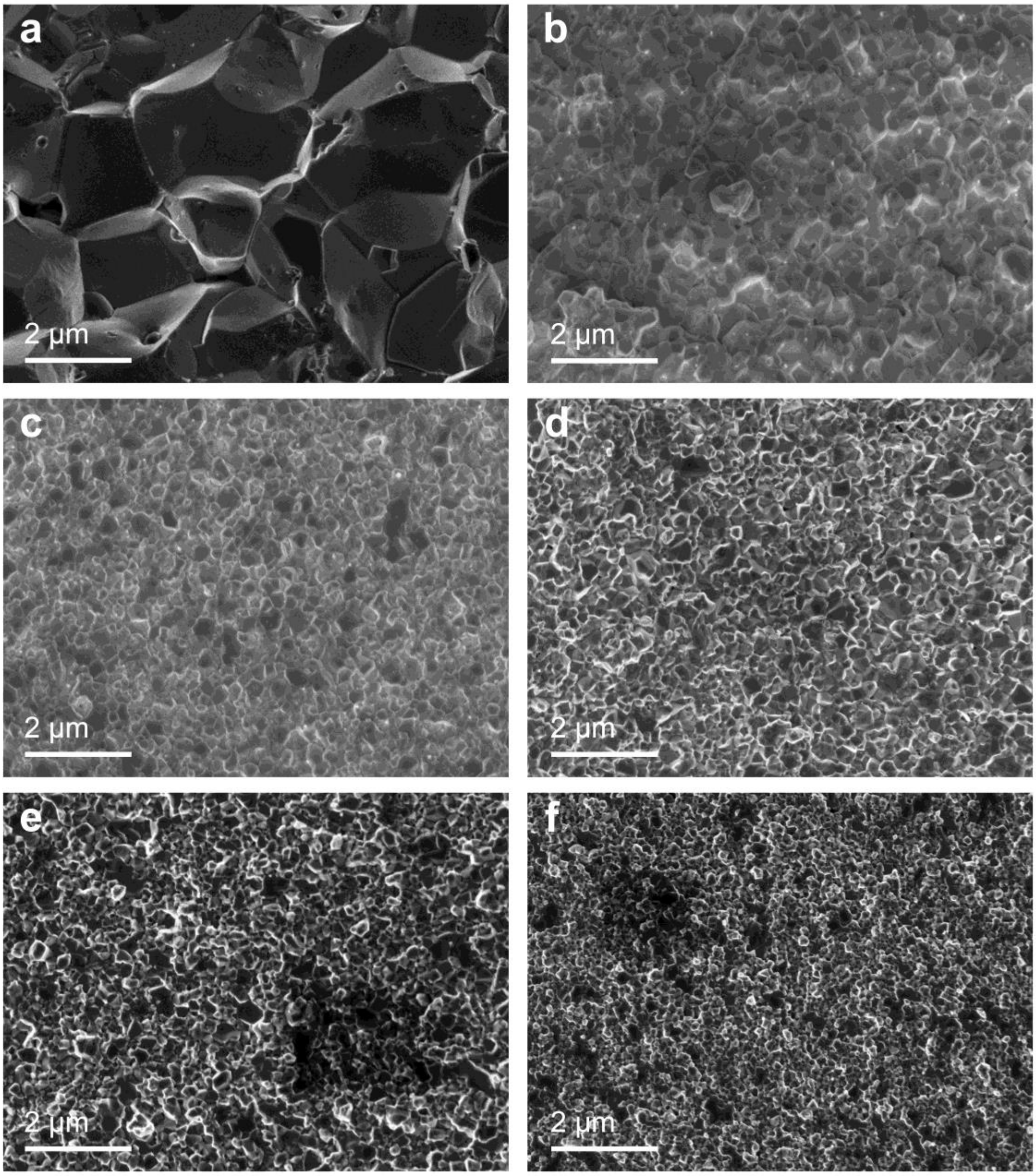

Supplementary Figure 4. SEM images of the hot-pressed $ZrCoBi_{1-x-y}Sb_ySn_x$ with different doping and alloying concentration. Undoped ZrCoBi (a), $ZrCoBi_{0.95}Sn_{0.05}$ (b), $ZrCoBi_{0.90}Sn_{0.10}$ (c), $ZrCoBi_{0.85}Sn_{0.15}$ (d), $ZrCoBi_{0.80}Sn_{0.20}$ (e), and $ZrCoBi_{0.65}Sb_{0.15}Sn_{0.20}$ (f). The grain size of the hot-pressed samples decreases with doping and alloying concentration. The SEM image of $ZrCoBi_{0.65}Sb_{0.15}Sn_{0.20}$ (f) is taken from the sample after repeatedly measurement and ten times of thermal shock cycles form room temperature to 973 K.

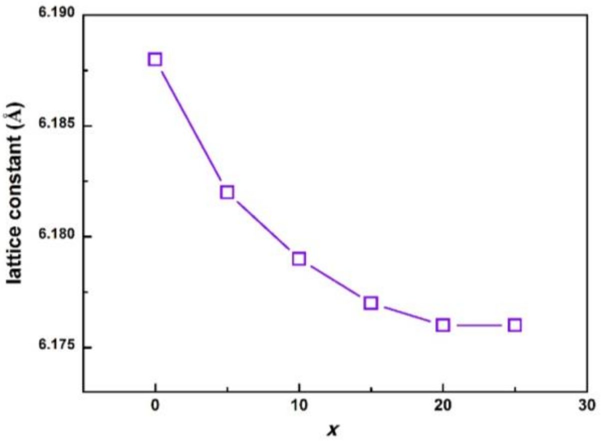

Supplementary Figure 5. The lattice constant of $ZrCoBi_{1-x}Sn_x$.

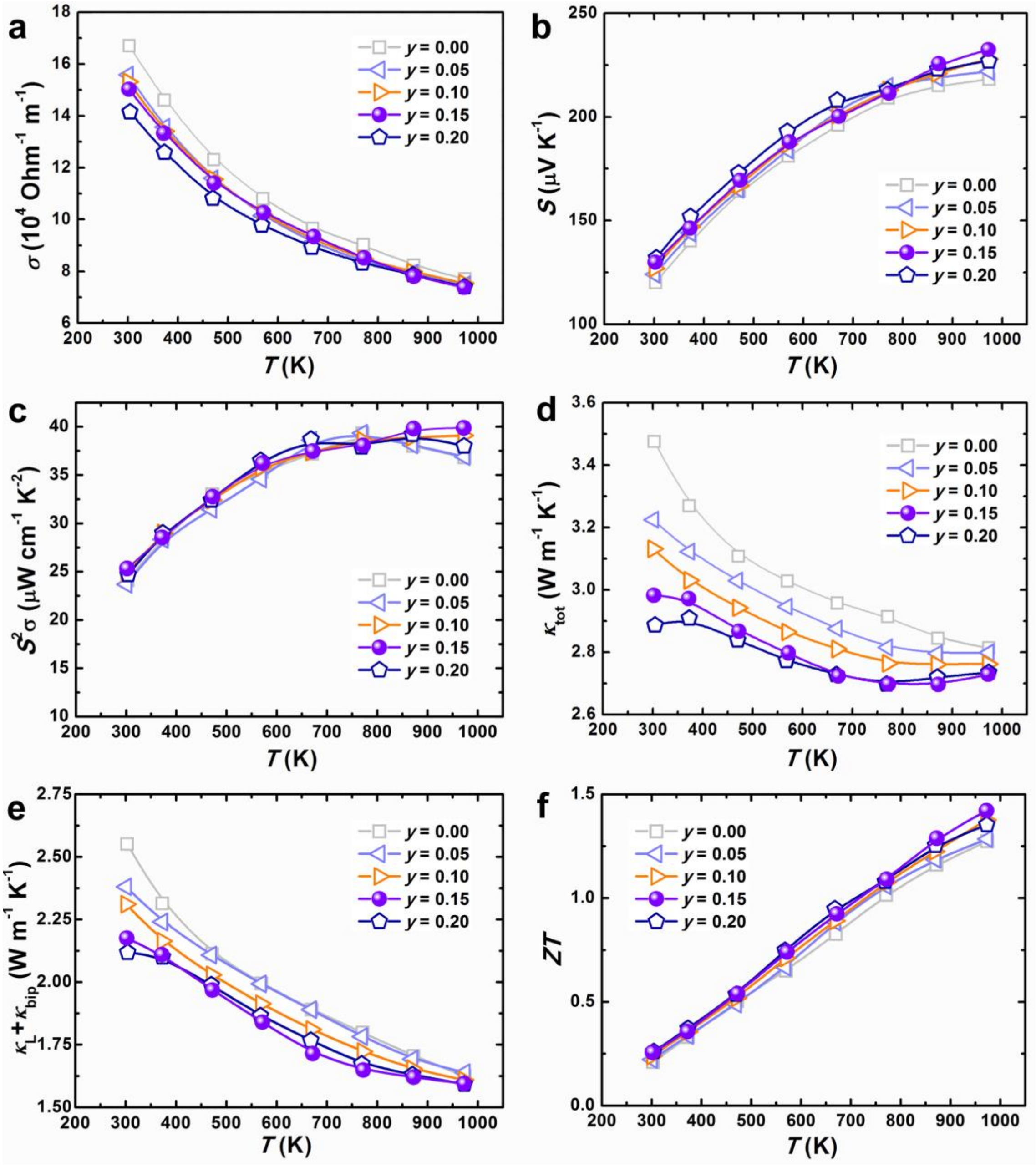

Supplementary Figure 6. Thermoelectric performance of $ZrCoBi_{0.80-y}Sb_ySn_{0.20}$ (y = 0, 0.05, 0.10, 0.15, 0.20). Electrical conductivity (a), Seebeck coefficient (b), power factor (c), total thermal conductivity (d), the summation of lattice and bipolar thermal conductivity (e), and *ZT* (f).

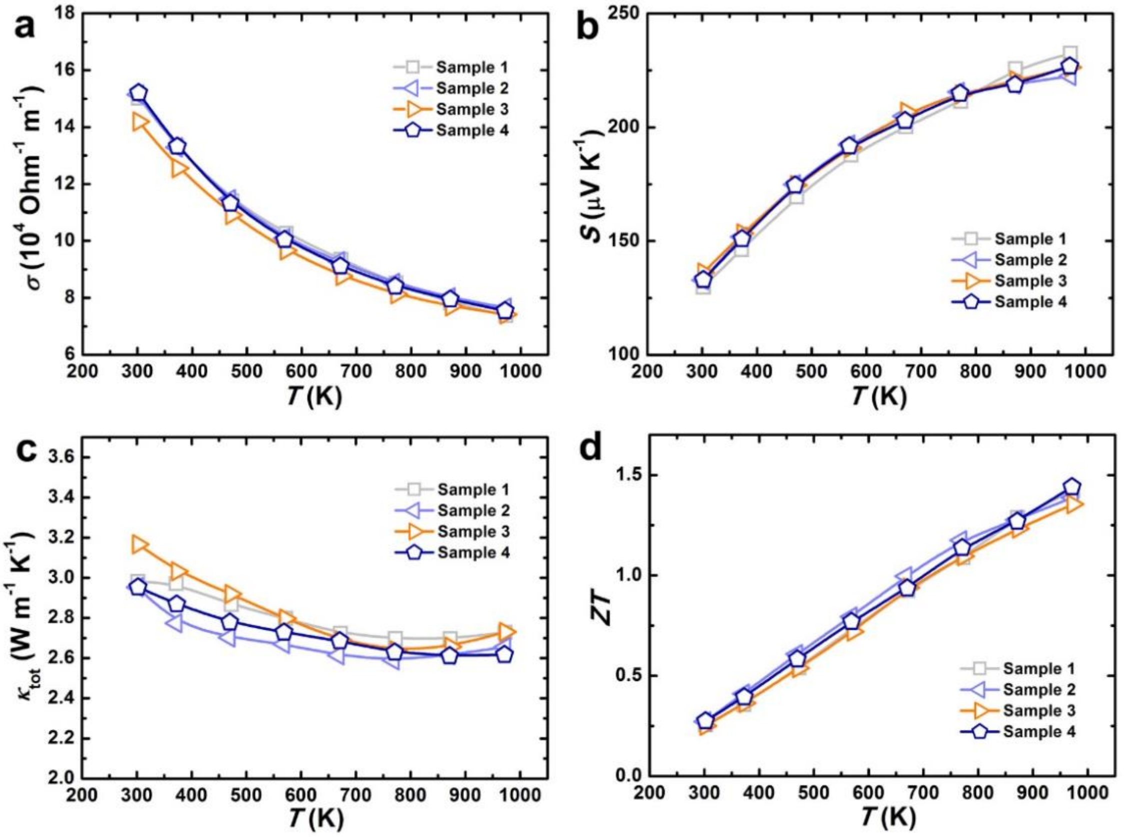

Supplementary Figure 7. Reproducibility of the thermoelectric performance of $ZrCoBi_{0.65}Sb_{0.15}Sn_{0.20}$. Electrical conductivity (a), Seebeck coefficient (b), total thermal conductivity (c), and *ZT* (f) of the four samples.

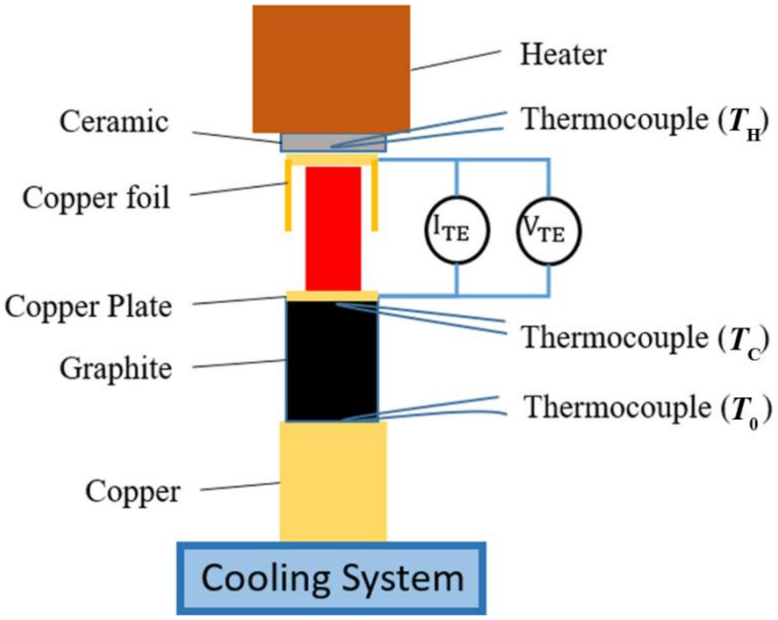

Supplementary Figure 8. Schematic diagram of efficiency and output power density measurement system for the single-leg device.

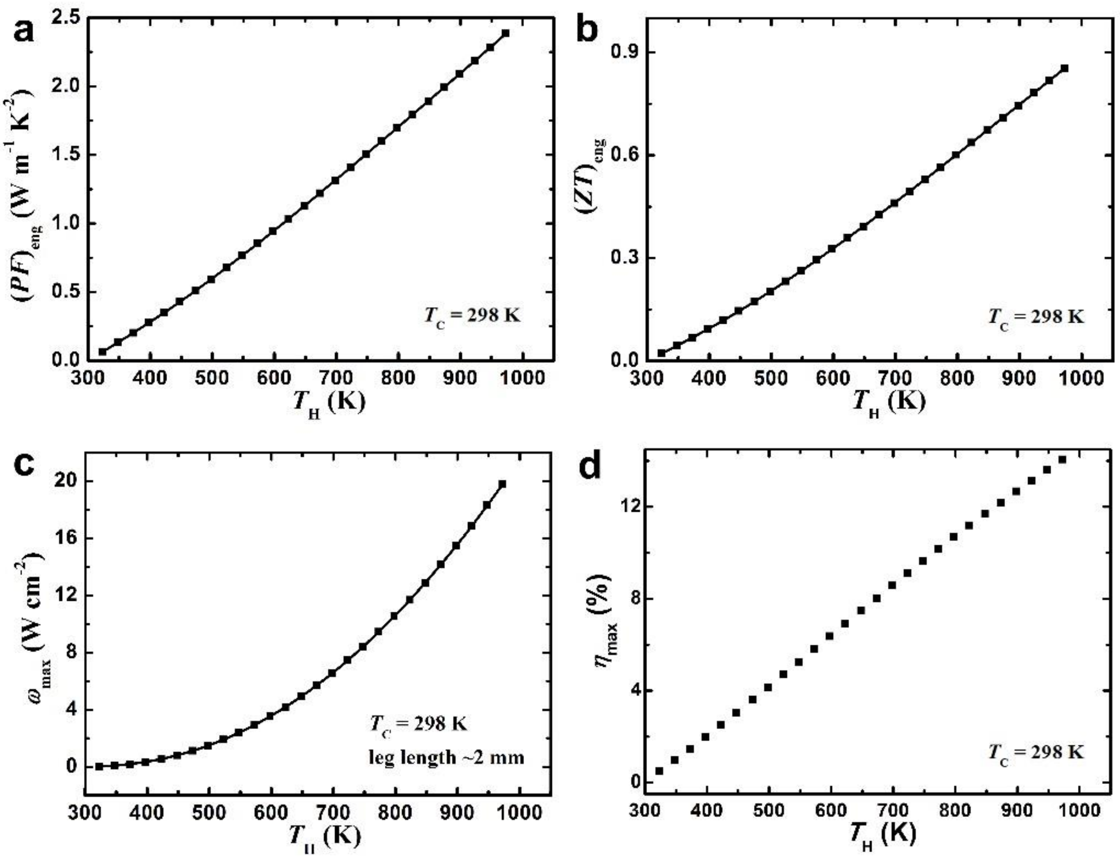

Supplementary Figure 9. Evaluating the heat recovery ability of $ZrCoBi_{0.65}Sb_{0.15}Sn_{0.20}$ by practical indicators. Calculated hot-side-temperature-dependent engineering power factor (a), engineering *ZT* (b), output power density (c) and conversion efficiency (d).

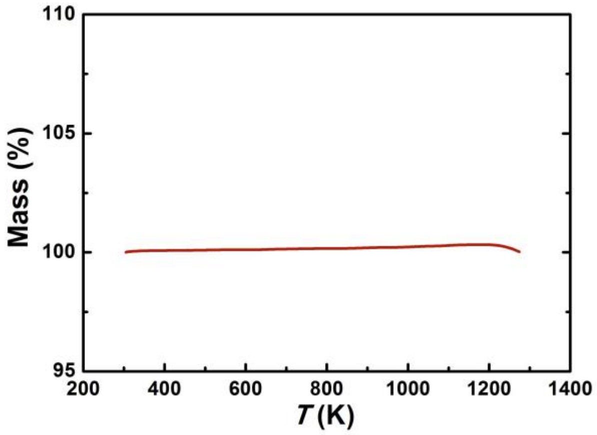

Supplementary Figure 10. Thermal stability test. Thermogravimetric analysis of $ZrCoBi_{0.65}Sb_{0.15}Sn_{0.20}$ in Ar atmosphere. No decomposition of the sample was observed up to 1273 K.

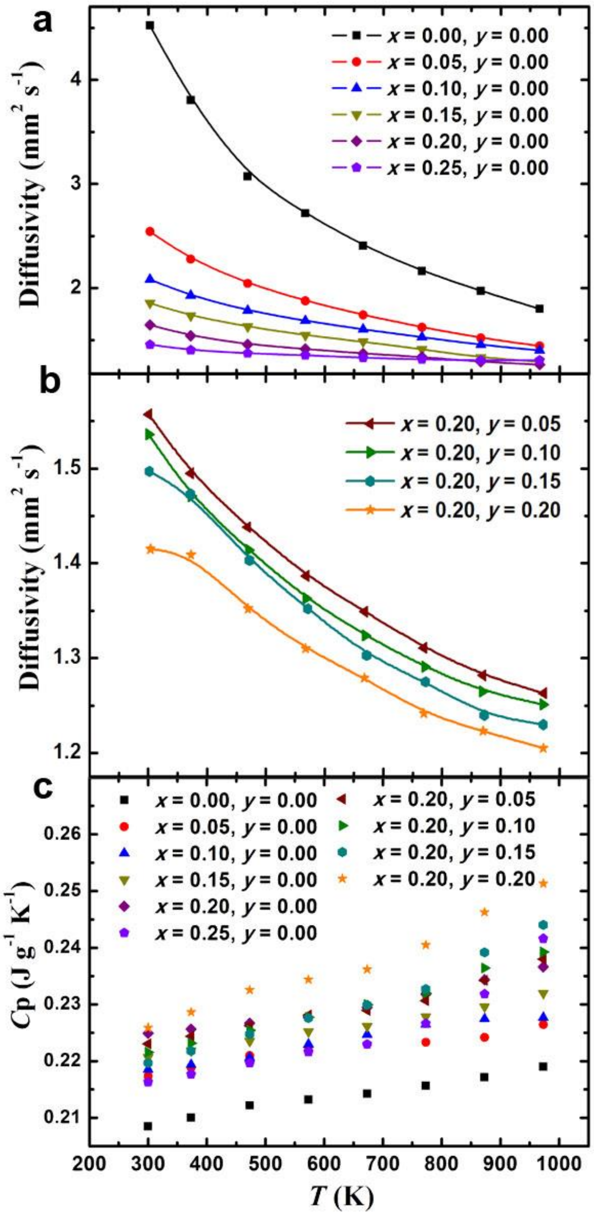

Supplementary Figure 11. Thermal transport properties. Temperature-dependent diffusivity (a, b) and specific heat (c) of $ZrCoBi_{1\text{-}x\text{-}y}Sb_ySn_x$.

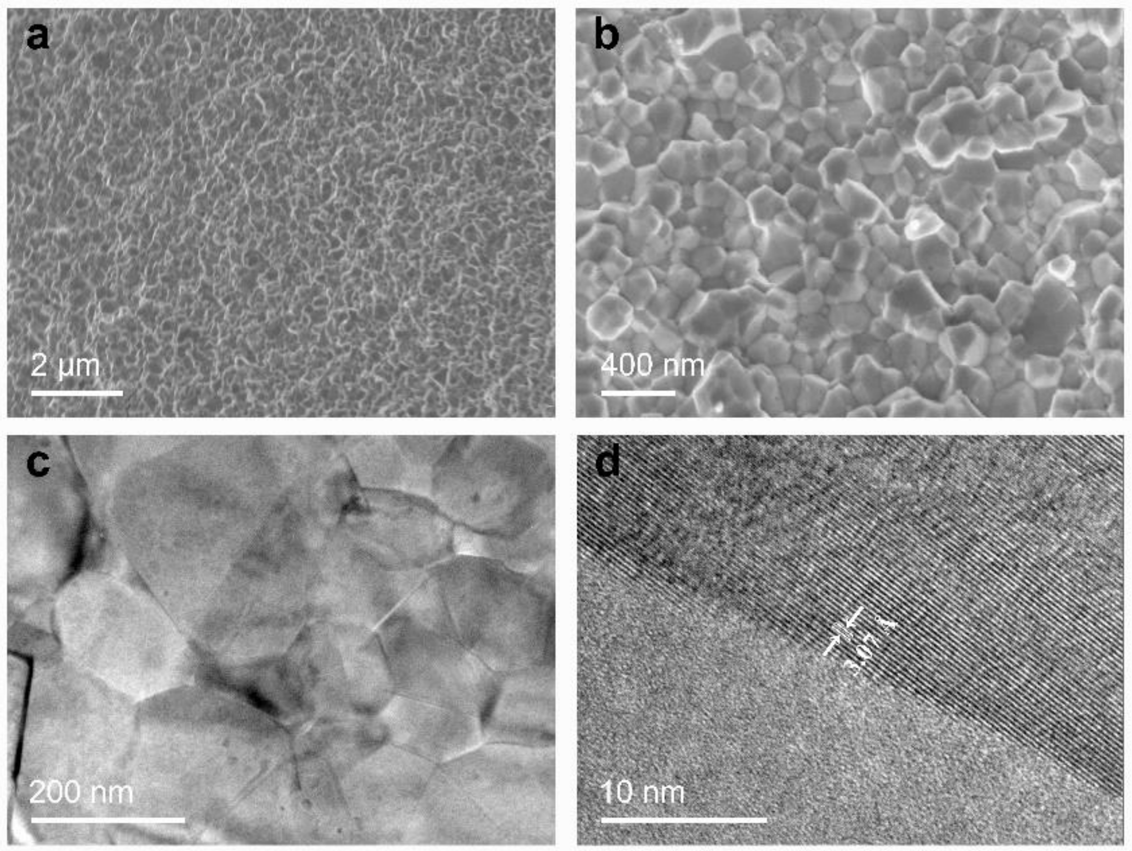

Supplementary Figure 12. The microstructure of the hot-pressed $ZrCoBi_{0.65}Sb_{0.15}Sn_{0.20}$. The SEM images (a-b) and TEM images (c-d) indicate the specimen is highly dense with an average grain size of ~200 nm.

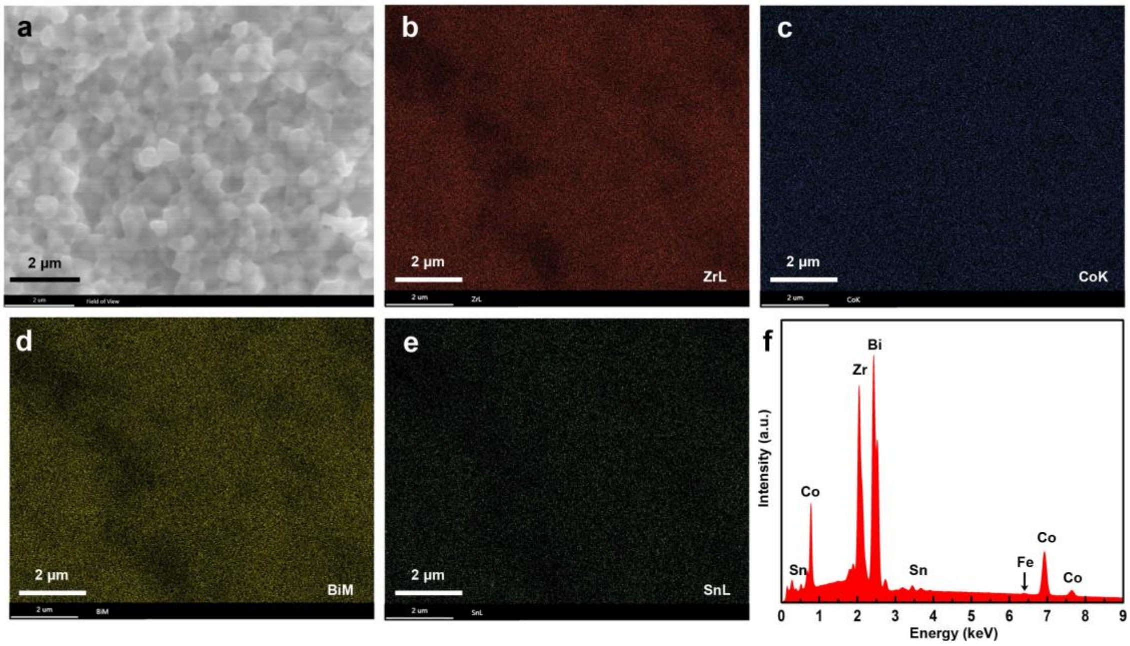

Supplementary Figure 13. The elemental mapping and chemical composition of the hot-pressed $ZrCoBi_{0.95}Sn_{0.05}$. The SEM image (a) and corresponding EDS elemental mapping of Zr (b), Co (c), Bi (d), Sn (e), and EDS spectrum (f).

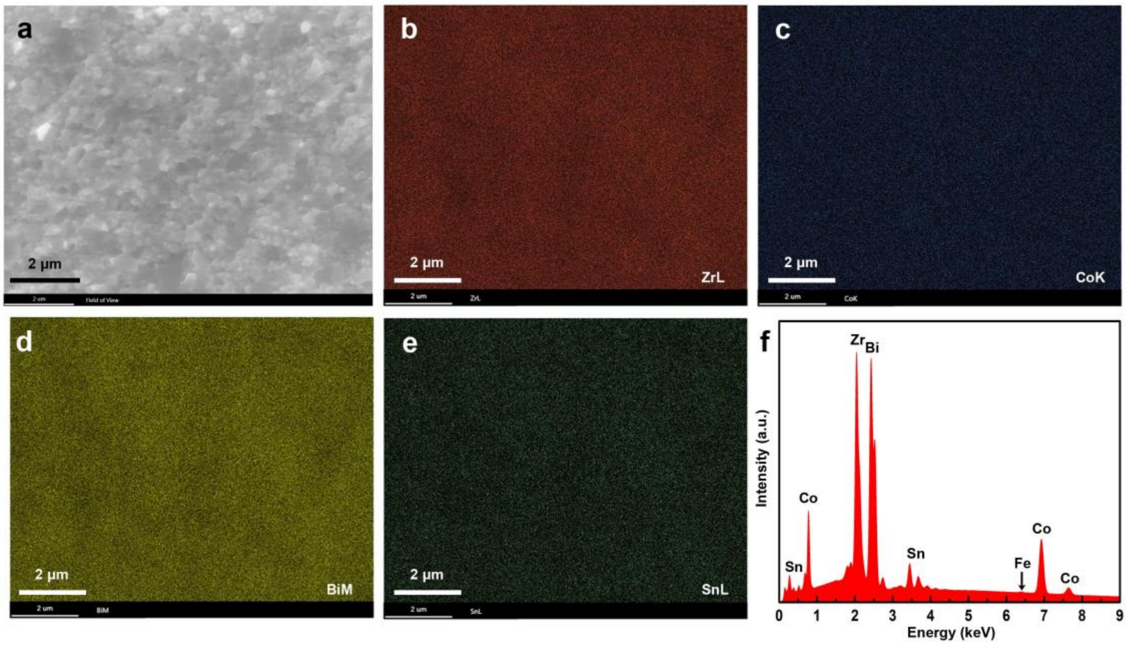

Supplementary Figure 14. The elemental mapping and chemical composition of the hot-pressed $ZrCoBi_{0.80}Sn_{0.20}$. The SEM image (a) and corresponding EDS elemental mapping of Zr (b), Co (c), Bi (d), Sn (e), and EDS spectrum (f).

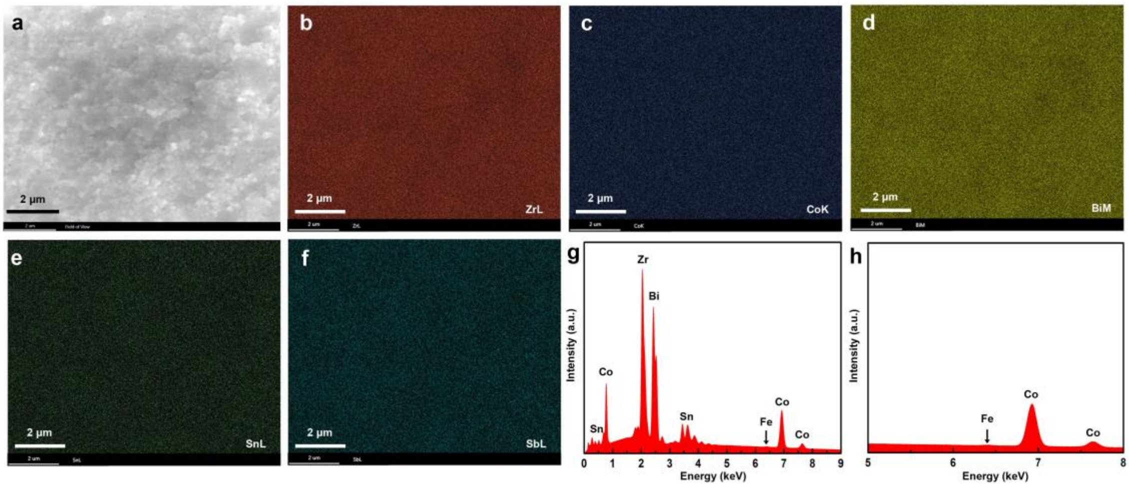

Supplementary Figure 15. The elemental mapping and chemical composition of the hot-pressed $ZrCoBi_{0.65}Sb_{0.15}Sn_{0.20}$. The SEM image (a) and corresponding EDS elemental mapping of Zr (b), Co (c), Bi (d), Sn (e), and Sb (f). EDS spectrum (g) and enlarge EDS spectrum focused on Fe peak (h). The analysis is taken from the sample after repeatedly measurement and ten times thermal shock cycles form room temperature to 973 K.

Supplementary Table

Supplementary Table 1. Elastic properties of undoped ZrCoBi and $ZrCoBi_{0.65}Sb_{0.15}Sn_{0.20}$ at room temperature. Longitudinal (v_L), transverse (v_t) and mean (v_m) sound velocity, Debye temperature (θ_D), Poisson ratio (υ_p) and Gruneisen parameter (γ).

	v_L (m s^{-1})	v_t (m s^{-1})	v_m (m s^{-1})	θ_D (K)	υ_p	γ
ZrCoBi	4613	2558	2849	314	0.278	1.64
$ZrCoBi_{0.65}Sb_{0.15}Sn_{0.20}$	4410	2468	2747	303	0.272	1.61
